# Stammering

**Published:** 1870-12

**Authors:** 


### STAMMERING

Is a nervous disease. One of the most inveterate stammerers became possessed with the idea that he would make a good play actor; the apparent absurdity of the thing attracted public attention, and his first appearance was greeted by one of the largest London audiences, and to the surprise of every one, he went through his part without one single blunder or hesitation. In this is the principle of cure in every conceivable case given for that purpose; and by knowing the principle involved, by having the key to one case, we have the key to all. This man's attention was so completely taken up, the nervous power was so strongly diverted to the memory of the words, only the ordinary amount went to the tongue.

A man stutters because the tongue is too full of nervous force; so full that the mind cannot restrain, regulate, guide. Most machines will work badly if worked too fast; every one knows that it is possible to be so much in a hurry to do a thing, that it cannot be done at all, giving rise to the saying, "More haste, worse speed." This is but a stammering of the hands or fingers. All that is necessary to a cure in any case, is to obtain self-control. There is an excessive run of nerve power to the tongue; divert a portion of it in another direction, and the cure is certain in every case. For example, when the stammerer attempts to speak, he should endeavor to do something else at the same time, say with foot or finger. Let him stamp his foot on the ground at the same time that he utters each syllable, and stammering is impossible.

The most inveterate stutterer reads as distinctly as any man, although we do not remember to have seen this fact noticed; it is because part of the attention is attracted to the notice of the words; it answers the same purpose to tap the finger against some object at each utterance. In this way the most obstinate case can be permanently cured in a few months. In the case of children, it should never be remarked upon; some parents get angry, and begin to scold or threaten, and even punish the child on the instant of its beginning to stammer; this only aggravates the case, because it confuses and alarms, and is fixed for life. Better do all possible to encourage, to compose, to give confidence; ridicule deepens and fixes the habits. Many other nervous affections, twitches, motions, noises from the nose, mouth, lungs, &c., are aggravated and deepened by harshness, as well as by ridicule, and become life-long blemishes.

